# Measuring Anxiety in Patients With Early-Stage Parkinson's Disease: Rasch Analysis of the State-Trait Anxiety Inventory

**DOI:** 10.3389/fneur.2019.00049

**Published:** 2019-02-13

**Authors:** Hui-Jun Yang, Joon-Ho Ahn, Jungsun Lee, Won Kee Lee, Jiho Lee, Yangho Kim

**Affiliations:** ^1^Department of Neurology, Ulsan University Hospital, University of Ulsan College of Medicine, Ulsan, South Korea; ^2^Department of Psychiatry, Ulsan University Hospital, University of Ulsan College of Medicine, Ulsan, South Korea; ^3^Department of Psychiatry, Asan Medical Center, University of Ulsan College of Medicine, Seoul, South Korea; ^4^Medical Research Collaboration Center, Kyungpook National University Hospital and School of Medicine, Kyungpook National University, Daegu, South Korea; ^5^Department of Occupational and Environmental Medicine, Ulsan University Hospital, University of Ulsan College of Medicine, Ulsan, South Korea

**Keywords:** Parkinson's disease, item response theory, State-Trait Anxiety Inventory, anxiety, validity, reliability, Rasch analysis

## Abstract

The State-Trait Anxiety Inventory (STAI), composed of two 20-item subscales (STAI-state and STAI-trait), has been increasingly used to assess anxiety symptoms in patients with Parkinson's disease (PD). However, the clinimetric attributes of the STAI under the statistical framework of the item-response theory (IRT) have not been fully elucidated within this population to date. We performed an IRT-based Rasch analysis of the STAI outcomes of patients with *de novo* PD from the Parkinson's Progression Markers Initiative database. The unidimensionality, Rasch model fit, scale targeting, separation reliability, differential item functioning, and response category utility of the STAI were statistically evaluated. A total of 326 (209 males, 117 females) patients without cognitive dysfunction were enrolled in our study. The original versions of the STAI-state and STAI-trait had acceptable separation reliability but lacked appropriate response category functioning, exhibited scale off-targeting, and several items demonstrated poor fit to the Rasch model. The response categories were reduced from four to three, and the rescored three-point TASI-trait demonstrated a marked improvement in clinimetric properties without a significant impact on unidimensionality and separation reliability. The rescored three-point version of the STAI-state required the additional removal of four misfitting items in order to improve the Rasch model fit. To our knowledge, this is the first study to assess the measurement properties based on the IRT of the STAI in patients with PD. Our Rasch analysis identified the components requiring possible amendments in order to improve the clinimetric attributes of the STAI.

## Introduction

Anxiety is a prevalent non-motor symptom that affects 12–57% of patients with Parkinson's disease (PD) (1–3). The structural evaluation of anxiety is crucial for the effective management of PD; therefore, the importance of the reliability and validity of the clinical rating scale used to assess the anxiety symptoms in patients with PD has been highlighted (3–5).

The classical test theory (CTT) is a popular statistical framework for evaluation of the reliability and validity of questionnaires, patient-reported outcomes, and rating scales in health-care studies (6–8). The CTT hypothesizes that an observed score is the sum of a true score (error-free score) and measurement error, and the true score in the CTT is estimated based on the mean value of repeatedly measured observed scale scores (6, 7). The CTT is relatively easy to interpret and has thus far been applied in the standardization of the diverse clinical rating scales; however, there are several known limitations of the CTT. Shortcomings of the CTT include dependency between the clinimetric properties of the rating scale and the patients' responses, an ordinal level of measurement rather than interval, and a lack of statistical assessment for polytomous response category function (8–10). The modern test theory or item response theory (IRT) was developed to complement the above limitations of the CTT (5, 7). According to the statistical framework of the IRT, the item and patient statistics are derived in a mutually independent manner (6, 7). The IRT calculates the probability of the patient's response to any particular item and converts the probability into a logit score that has properties of an interval scale (6, 9, 10). Moreover, the IRT can administer a series of fit statistics, average measures, and step calibration of the response categories, consequently allowing for the diagnostic analysis of polytomous category utility (10–13). The Rasch analysis, which is a widely used one-parameter IRT model approach, has previously been successfully utilized to validate several anxiety rating scales among patients with PD, including the Hospital Anxiety and Depression Scale-Anxiety subscale (HADS-A), Hamilton anxiety rating scale (HARS), and Beck Anxiety Inventory (BAI) (5, 14).

The State-Trait Anxiety Inventory (STAI), which was first developed in the 1960s by Spielberger et al. was designed to rate overall adult anxiety and is formulated as a four-point Likert scale (STAI “Form X”) (15). A revision of the STAI, which reduced the overlap with depression and placed emphasis on better described state and trait anxiety factors, was published in 1983 (STAI “Form Y”) (16). The STAI was applied in prior studies for quantification of anxiety symptoms in patients with PD (4, 5), and its reliability and validity based on the CTT have been demonstrated (17, 18). However, none of the previous studies have performed an IRT-based analysis of the clinimetric attributes of the STAI in patients with PD (3, 4). Moreover, concerns have been raised with regard to the suitability of the polytomous response structure including a multi-point Likert scale in the application in older adults, such as patients with PD (4, 19, 20). While the determination of the response category options is typically considered a priori in the CTT, Rasch analysis provides diagnostic statistics to evaluate if the empirically-determined polytomous response categories function as intended, and alternative rescoring can improve the overall measurement properties of scale (5, 10, 12).

The present study aims to address the measurement properties of the STAI in PD patients without cognitive dysfunction by conducting Rasch analysis with demonstration of the Rasch model fit, separation reliability, differential item functioning (DIF), and response category function of a multi-point Likert scale of STAI.

## Methods

The Parkinson's Progression Markers Initiative (PPMI) study is 5-years international multi-center prospective investigation that aims to identify potential biomarkers of disease progression using clinical, imaging, and biospecimen data from newly diagnosed patients with PD (21). The detailed design and procedure of the PPMI study have been previously published elsewhere (2, 21, 22). The study protocol was approved by the institutional review boards of all participating centers, and each patient was required to provide written informed consent before study participation. In our study, patients with untreated PD at baseline who met the selection criteria were recruited from the PPMI online database (http://www.ppmi-info.org/data), and the final database was accessed on March 31, 2018. The selection criteria in this Rasch analysis include the following: (1) complete baseline data of the clinical assessments including the STAI, Montreal cognitive assessment (MoCA), and dopamine transporter scans (21, 22); (2) MoCA total score above the cut-off value of mild cognitive impairment (MoCA score of ≥26) (23); and (3) modified Hoehn and Yahr (H-Y) stage ≤2.

STAI “Form Y,” consisting of two 20-item subscales, including the STAI state subscale (STAI-state) and STAI trait subscale (STAI-trait), was employed to quantify a range of anxiety symptoms associated with the disease (16). The STAI-state is used for measurement of temporary anxiety symptoms due to specific situations or particular objects. Responses in the STAI-state are formulated as a four-point Likert scale with the following category options: category 1 for “not at all,” category 2 for “somewhat,” category 3 for “moderately so,” and category 4 for “very much so.” The STAI-trait addresses innate and relatively stable personal tendencies to experience anxiety symptoms. Responses in the STAI-trait have the following four category options: category 1 for “almost never,” category 2 for “sometimes,” category 3 for “often,” and category 4 for “almost always.” Some *anxiety-absent* items (STAI-state items 1, 2, 5, 8, 10, 11, 15, 16, 19, and 20; STAT-trait items 21, 23, 26, 27, 30, 33, 34, 36, and 39) are negatively keyed, and these items are reverse-coded according to the instructions (16, 21). The score range for both the STAI-state and the STAI-trait is 20–80 points, and a higher score indicates a greater degree of anxiety. The disease severity of the patients was globally addressed by the H-Y staging and Movement Disorders Society Unified Parkinson's Disease Rating Scale (MDS-UPDRS) parts I, II, and III. The 15-item Geriatric Depression Scale was used to evaluate the intensity of depressive symptoms, and the MoCA was employed to evaluate overall cognitive function (22).

The analyses of internal consistency and convergent validity based on the CTT were presented using IBM SPSS version 19 (IBM corporation, Somers, NY, USA). For internal consistency, Cronbach's α-coefficient (should be >0.70) and item-total correlation (should be >0.30) were calculated (3, 4, 8). Convergent validity was assessed using the Spearman rank-order correlation with item 4 (anxiety symptoms) of the MDS-UPDRS part I, and the Spearman correlation coefficients of *r*_S_ >0.40 was considered to indicate a moderate or greater correlation (7, 8).

The unidimensionality, Rasch model fit of items, person separation reliability, scale targeting, DIF, and response category utility were statistically evaluated by Rasch analysis (Supplementary Figure 1) based on Andrich's rating scale model using WINSTEPS version 4.0.1 (Winsteps Inc., Chicago, IL, USA) (10). Unidimensionality of the rating scale was assessed by a principal component analysis of the residuals (PCAR) wherein the Rasch factor is extracted. If the variance as explained by the Rasch factor was ≥40%, it was considered to support unidimensionality (6, 24). An eigenvalue for the first or second residual variances of ≥3.0, or ≥10% of variance as explained by the first or second contrast, were thought to demonstrate the possibility of multidimensionality (25–27). The Rasch model fit indicating internal scale validity was investigated based on the infit (the information-weighted fit) and outfit (the outlier-sensitive fit) statistics (24). The mean-square (MnSq) value of infit and outfit was expected to be 1, and the acceptable range of the MnSq value was 0.5–1.5 (9, 13, 28). The measurement reliability of the STAI-state and STAI-trait were assessed by the person separation reliability (PSR), which measures the capacity of the discrimination among the patient groups with different levels of anxiety (7). The tentative criterion value of the PSR was 0.8, which indicates that the scale was able to discriminate the study patients into three strata of anxiety severity (i.e., mild, moderate, or severe) (22, 28). A person-item distribution map (Wright map) was used to visually inspect the STAI item measurement range with respect to the patient symptom severity distribution. The range of patient measures and item measures were displayed on the left and right side of the map, respectively. Test items with a higher logit score were located on the top of the map and considered measuring higher level of symptoms, while items on the bottom of the map were in the area of relatively lower patient anxiety. The difference in the person-item mean logit was used to address overall targeting of the rating scale (6, 9, 24). DIF was analyzed to determine whether STAI items function differently in relation to key demographic variables including gender (male vs. female) and age (aged < 60 vs. aged ≥60 years as older) (7, 12). DIF was considered to be significant if DIF contrast above 0.60 logits difference (29, 30). The utility of each response category of the four-point Likert scale was analyzed by the probability curves, outfit of the residual MnSq value, patient count, average measures, and step calibration for each response category (10, 31). The probability curves of the multi-point response options present the likelihood of patients selecting a certain response option on the STAI at various levels of the anxiety. Ordered thresholds on the probability curves, a minimum patient count of 10 in each category, an outfit MnSq value of < 2.0, the hierarchical monotonic increase in average measures and step calibrations were required for appropriate category function (31, 32).

## Results

A total of 326 patients with untreated PD including 209 men and 117 women (age range, 34–85 years) were enrolled. The median H-Y stage was 2. The mean score of the STAI-state was 33.1 (range, 0–45) and that of the STAI-trait was 32.4 (range, 0–45). Table 1 presents the demographic and clinical data of the study patients.

**Table 1:** Demographic and clinical characteristics of 326 patients with Parkinson's disease.

**Characteristics**	**Number**	**Percentage (%)**	**Mean**	**SD**
Age, years			60.8	9.7
Gender				
Male	209	64.1		
Female	117	35.9		
Education, years			15.7	3.1
Disease duration, months			6.0	3.6
Hoehn and Yahr stage			1.6	0.5
Stage 1	147	45.1		
Stage 2	179	54.9		
MDS-UPDRS part 1			5.5	4.2
MDS-UPDRS part 2			6.0	4.3
MDS-UPDRS part 3			20.2	8.4
MoCA score			28.1	1.3
STAI-state score			33.1	10.6
STAI-trait score			32.4	9.6
GDS-15 score			5.3	1.4

*SD, Standard Deviation; MDS-UPDRS, Movement Disorder Society—Unified Parkinson's Disease Rating Scale; STAI-state, State-Trait Anxiety Inventory state subscale; STAI-trait, State-Trait Anxiety Inventory trait subscale; MoCA, Montreal Cognitive Assessment; GDS-15, Geriatric Depression Scale 15-item short form*.

With regard to internal consistency according to the CTT, the Cronbach's α coefficient of the STAI-state and STAI-trait was 0.932 and 0.922, respectively, both of which were higher than the threshold value of 0.70. The item-total correlation score for the STAI-state and STAI-trait was 0.377–0.764 and 0.446–0.726, respectively, and all items met the criterion for internal consistency (item-total correlation of >0.30). Concerning the convergent validity, question four of the MDS-UPDRS part I exhibited moderate or greater correlation with the STAI-state (*r*_S_ = 0.427, *p* < 0.000) and STAI-trait (*r*_S_ = 0.523, *p* < 0.000).

**Figure 1:**
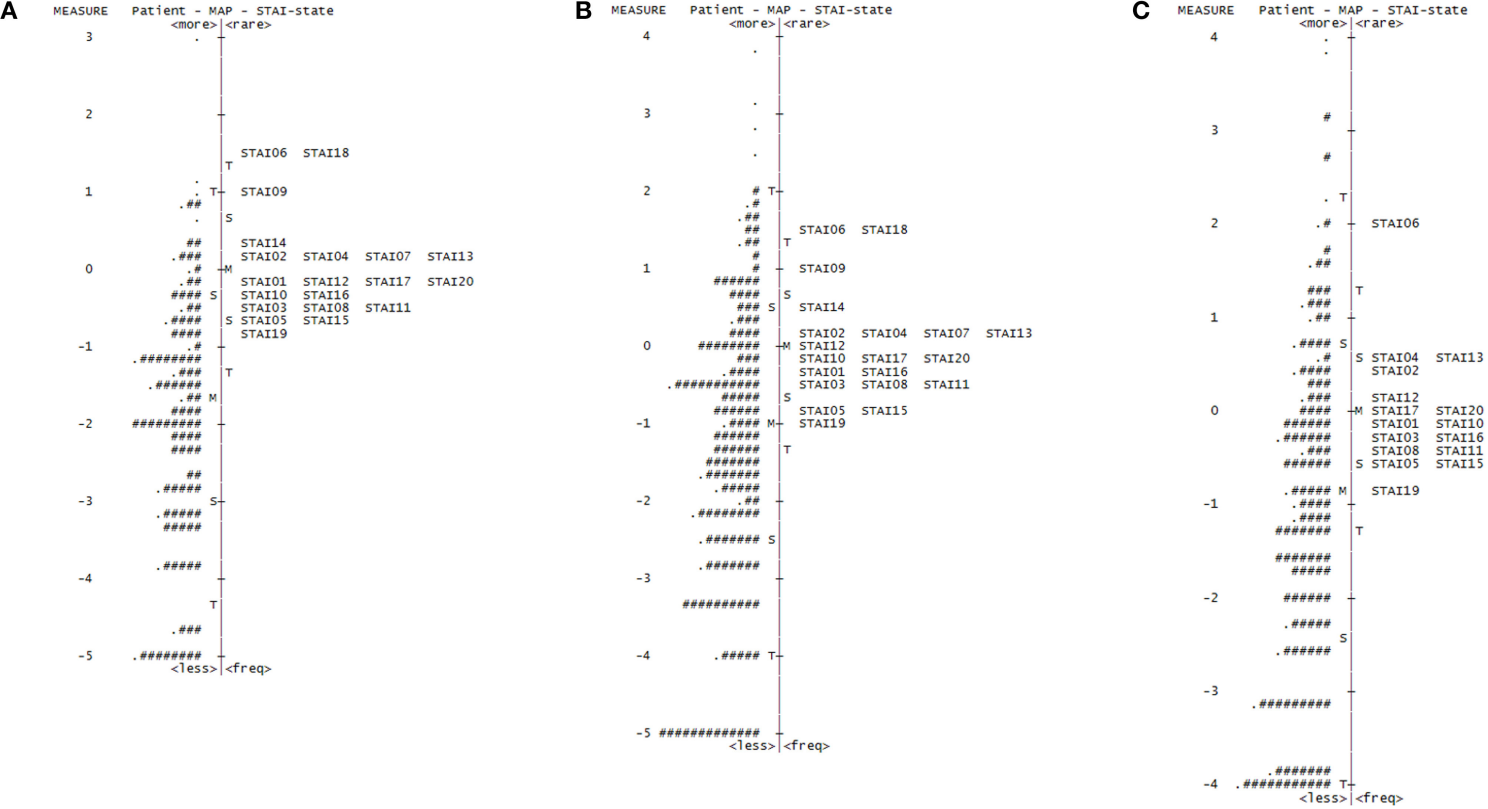
Person-item distribution plot (Wright map) of the STAI-state. Positive scores demonstrate higher levels of anxiety. M, mean of person or item distribution; S, 1 standard deviation from the mean; T, 2 standard deviations from the mean. **(A)** Wright map for the original 20-item State-Trait Anxiety Inventory state (STAI-state) subscale (four-point scale). “#” Indicates three persons and “.” indicates one to two persons. **(B)** Wright map for the rescored 20-item STAI-state (three-point scale). “#” Indicates two persons and “.” indicates one person. **(C)** Wright map for the shortened 16-item STAI-state (three-point scale). “#” Indicates three persons and “.” indicates one to two persons.

**Figure 2:**
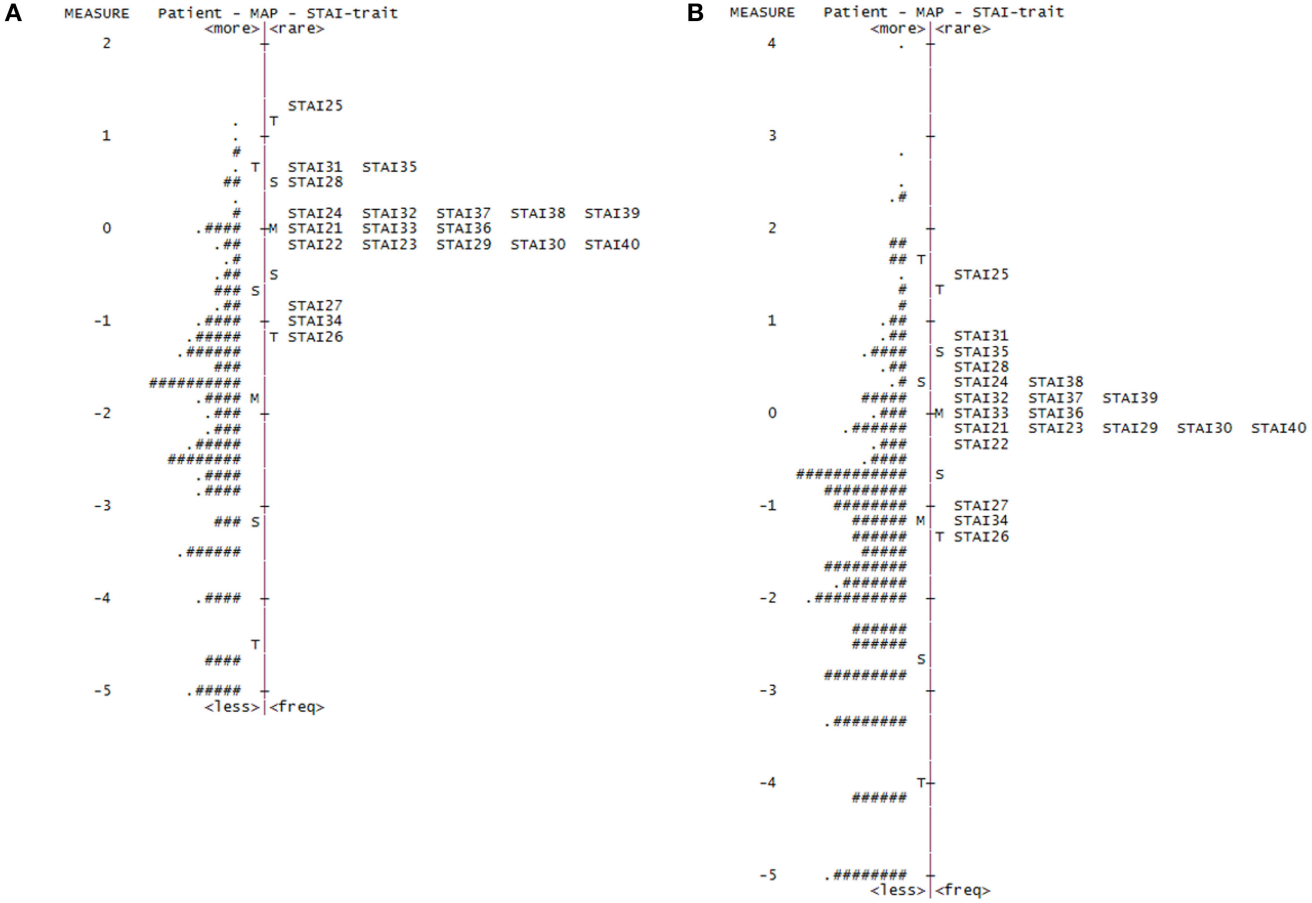
Person-item distribution plot (Wright map) of the STAI-trait. Positive scores demonstrate higher levels of anxiety. M, mean of person or item distribution; S, 1 standard deviation from the mean; T, 2 standard deviations from the mean. **(A)** Wright map for the original State-Trait Anxiety Inventory trait (STAI-trait) subscale (four-point scale). “#” Indicated three persons and “.” indicates one to two persons. **(B)** Wright map for the rescored STAI-trait (three-point scale). “#” Indicates two persons and “.” indicates one person.

Our initial Rasch analysis indicated that the acceptable separation reliability of the STAI-state and STAI-trait as assessed by PSR were 0.83 and 0.83, respectively, both of which were higher than the tentative criterion value of 0.80 (Table 2). However, three STAI-state items (item 9, 14, and 18) failed to meet the criterion value (between 0.5 and 1.5) of the infit or outfit MnSq values, indicating that the actual responses to these items did not match the expectation of the Rasch model (Table 2). DIF for gender was found for items 9 and 18 (greater in females). None of the STAI-state items exhibited age-related DIF. In the STAI-trait, four items (namely items 24, 32, 35, and 38) failed to demonstrate an acceptable outfit MnSq value for the Rasch model fit. While all STAI-trait items were free from DIF for age, items 25 and 35 exhibited gender-related DIF that was more severe in females. According to the person-item distribution map with the mean logit for patients set to 0, the mean logit for the STAI-state item was −2.03, indicating that item measurement range was highly distributed compared to the severity of anxiety symptoms in the study patients (Figure 1; Table 2). The person-item map (Figure 1) also displayed the hierarchy of the STAI-state items. Item 6 “I feel upset” and item 18 “I feel confused” were the hardest to endorse by patients with PD, and item 19 “I feel steady” the easiest. Similarly, item measurement distribution of the STAI-trait was targeted relatively higher than the degree of anxiety symptoms in the patient group with the mean difference in logit for items of −2.09 (Figure 2; Table 2). Figure 2 indicated that STAI-trait item 25 “I feel like a failure” was the hardest to endorse, whereas item 26 “I feel rested” was the easiest.

**Table 2:** Psychometric validation-related statistics based on the Rasch model for the State-Trait Anxiety Inventory state and the State-Trait Anxiety Inventory trait subscales.

**Rasch statistics of the clinical scales**	**Original 20-item scale (four-point)**	**Rescored 20-item scale (three-point)**	**Shortened 16-item scale (three-point)**
**STAI-STATE**			
Rasch factor eigenvalue (variance %)	17.17 (46.2)	18.62 (48.2)	14.38 (47.3)
1st contrast eigenvalue (variance %)	2.90 (7.8)	2.91 (7.5)	2.88 (9.5)
2nd contrast eigenvalue (variance %)	1.81 (4.9)	1.92 (5.0)	1.56 (5.1)
Point-measure correlation, range	0.35~0.75	0.40~0.78	0.48~0.80
Items with infit MnSq <0.5 or >1.5 (%)	2 (10.0%)	0 (0.0%)	0 (0.0%)
Items with outfit MnSq <0.5 or >1.5 (%)	3 (15.0%)	4 (20.0%)	0 (0.0%)
Person separation reliability (index)	0.83 (2.20)	0.85 (2.35)	0.84 (2.27)
Item separation reliability (index)	0.97 (6.13)	0.98 (6.67)	0.98 (6.26)
Mean logit of the patient (standard deviation)	−2.03 (1.70)	−1.39 (1.85)	−1.22 (1.92)
**STAI-TRAIT**			
Rasch factor eigenvalue (variance %)	14.79 (42.5)	16.04 (44.5)	
1st contrast eigenvalue (variance %)	2.86 (8.2)	2.96 (8.2)	
2nd contrast eigenvalue (variance %)	1.62 (4.6)	1.51 (4.4)	
Point-measure correlation, range	0.46 ~ 0.72	0.50 ~ 0.73	
Items with infit MnSq < 0.5 or >1.5 (%)	0 (0.0%)	0 (0.0%)	
Items with outfit MnSq < 0.5 or >1.5 (%)	4 (20.0%)	0 (0.0%)	
Person separation reliability (index)	0.83 (2.18)	0.85 (2.35)	
Item separation reliability (index)	0.97 (5.29)	0.97 (5.68)	
Mean logit of the patient (standard deviation)	−2.09 (1.54)	−1.38 (1.69)	

*STAI-state, State-Trait Anxiety Inventory state subscale; MnSq, mean squares; STAI-trait, State-Trait Anxiety Inventory trait subscale*.

Concerning the response option utility, the category probability curves exhibited an ordered threshold for each response category in both the STAI-state and STAI-trait (Supplementary Figure 2). However, the outfit MnSq value of category 4 in the STAT-state exceeded the criterion value of 2.0. Category 4 of the STAI-trait also failed to meet the criteria (outfit MnSq value of <2.0), and the average measure of category 3 (−0.20) in the STAI-trait was higher than that of category 4 (−0.29), not displaying a monotonic advance (Table 3). The response category functions of the four-point Likert scales were considered inappropriate, hence both of the STAI subscales were rescored by collapsing categories 3 and 4 (Supplementary Figure 2).

**Table 3:** Threshold ordering test statistics for the State-Trait Anxiety Inventory state and the State-Trait Anxiety Inventory trait subscales.

**Response categories**	**Count (%)**	**Average measure**	**Step calibration**	**Outfit MnSq**
**STAI-STATE (ORIGINAL 20-ITEM SCALE)**				
Category 1 (not at all)	3501 (64)	−2.62	None	0.93
Category 2 (somewhat)	2003 (12)	−1.15	−1.47	0.83
Category 3 (moderately so)	766 (10)	−0.16	0.22	0.95
Category 4 (very much so)	249 (8)	0.10	1.25	2.81
**STAI-STATE (RESCORED 20-ITEM SCALE)**				
Category 1	3501 (64)	−2.11	None	0.95
Category 2	2003 (12)	−0.42	−0.87	0.86
Category 3	1015 (16)	0.83	0.87	1.62
**STAI-TRAIT (SHORTENED 16-ITEM SCALE)**				
Category 1	3361 (50)	−2.03	None	0.96
Category 2	2239 (33)	−0.35	−0.96	0.85
Category 3	1135 (17)	0.91	0.96	1.34
**STAI-TRAIT (ORIGINAL 20-ITEM SCALE)**				
Category 1 (almost never)	3512 (54)	−2.66	None	0.98
Category 2 (sometimes)	2129 (33)	−1.39	−1.61	0.84
Category 3 (often)	705 (11)	−0.20	0.19	0.76
Category 4 (almost always)	173 (3)	−0.29	1.41	2.91
**STAI-TRAIT (RESCORED 20-ITEM SCALE)**				
Category 1	3512 (54)	−2.06	None	1.05
Category 2	2129 (33)	−0.63	−0.94	0.85
Category 3	878 (13)	0.79	0.94	1.31

*MnSq, mean squares; STAI-state, State-Trait Anxiety Inventory state subscale; STAI-trait, State-Trait Anxiety Inventory trait subscale*.

The rescored three-point version of the STAI-state indicated that the outfit MnSq value of four items (items 7, 9, 14, and 18) failed to demonstrate acceptable outfit MnSq values, while the infit MnSq value for all rescored STAI-state items met the criteria. We also found notable gender-related DIF in items 9 and 18. After the above four misfitting items were removed, the infit, and outfit MnSq values of all 16 items were acceptable and free from DIF for gender and age. The revised 16-item STAI-state on a three-point Likert scale exhibited unidimensionality and the PSR (0.84) met the criteria. The range of item measurement was altered to better fit the distribution of the patients' symptoms compared to that prior to revision, which was confirmed by a significant improvement in the mean logit from −2.08 before revision to −1.39 after revision (Figure 1, Table 2). Category function analysis of the revised three-point STAI-state with 16 items indicated that the outfit MnSq value for all response categories was <2.0 and the average measure monotonically increased (Table 3).

The rescored three-point version of the STAI-trait presented apparent improvements in Rasch model fit, as all items of the rescored three-point STAI-trait had infit and outfit MnSq values between 0.5 and 1.5, and therefore all 20 items were retained (Table 2). A PCAR after the Rasch factor extraction ensured that the 20 items of the rescored STAI-trait were of a unidimensional construct. The PSR of the rescored STAI-trait (0.85) was adequate, and the category function analysis result was also satisfactory (Table 3). Only one item (item 35) exhibited a gender DIF contrast of −0.67 and no items exhibited age-related DIF. The person-item map indicated that the item measurement range after rescoring better reflected the distribution of the degree of the patients' symptoms (Figure 2, Table 2), that is supported by the improvement in the mean logit score from −2.09 prior to revision to −1.38 after revision.

We evaluated the reliability and validity of both of the revised subscales using the CTT. The rescored STAI-state (16 items) and the rescored STAI-trait (20 items) yielded Cronbach's α coefficients of 0.936 and 0.927, respectively, with item-total correlations in the range of 0.518–0.795 and 0.507–0.795. All values were higher than the thresholds. We confirmed a moderate or greater correlation with item 4 (anxiety symptoms) of the MDS-UPDRS part I for both the rescored STAI-state (16 items) and STAI-trait (20 items) according to the *r*_S_ values of 0.423 (*p* < 0.000) and 0.435 (*p* < 0.000), respectively.

## Discussion

This study explored the clinimetric attributes of STAI in non-demented *de novo* PD patients using the CTT and IRT (3, 7). Our result based on the CTT indicated that the STAI-state and STAI-trait displayed good internal consistency according to Cronbach's α coefficient and the item-total correlation. There was a moderate correlation with the MDS-UPDRS anxiety item, supporting the convergent validity in patients with PD. The current findings are in line with previous CTT studies in the PD population reporting that the STAI is significantly correlated with the HADS-A, HARS, and Geriatric Anxiety Inventory (GAI) (17, 18).

The initial Rasch analysis suggested that both the STAI-state and STAI-trait exhibited good separation reliability in patients with PD. However, the person-item distribution maps indicated that item difficulties of both STAI subscales were more highly distributed than the level of anxiety in the study patients, which indicated the inability of the questions to capture low level of anxiety and that the questions were off-targeted in PD (6, 9). The result of the present study was similar to a previous Rasch analysis in a PD cohort using other anxiety rating scales, including the HADS-A, BAI, and HARS, which were found to be more appropriate for patients with moderate or severe anxiety symptoms (5, 14). Off-targeting of the above scales was likely due to the fact that they were originally developed for assessment of anxiety in patients with more severe symptoms (4, 15, 16).

Testing with Rasch analysis also suggested possible issues in the function of the four-point polytomous response categories, and indicated that some of the items did not fit the Rasch model in the original STAI-state and STAI-trait (11, 27). To date, there have been several studies assessing the clinimetric properties of STAI in non-PD samples using Rasch analysis (33–37). Tenenbaum et al. found that the STAI-state and STAI-trait comprise some items that do not fit the Rasch model, and concluded that item deletion may be needed to refine the STAI-state and -trait scales in a non-PD population (33). Kaipper et al. found that both the 20-item STAI-state and the 20-item STAI-trait did not fit the Rasch model in the measurement of anxiety levels in surgical patients scheduled for elective operation. The authors reported that the shorter version of the STAI-state after removal of seven items and the shortened STAI-trait after removal of eight items have acceptable fit to the Rasch model (34). However, these two studies used the first version of the STAI (STAI “Form X”) in a relatively younger population (15, 16). In addition, the reduced number of rating scale items can result in decreased scale reliability, and some studies demonstrated that several shorter forms of the STAI were associated with reduced separation reliability (35, 38).

Recently, Davey et al. evaluated STAI “Form Y” in 322 ophthalmology patients (mean age ± *SD*: 61 ± 19 years) by Rasch analysis, indicating that response category reduction (combining categories 3 and 4) can improve the fit to the Rasch measurement model (36). Fernández-Blázquez et al. also proposed that recoding the four original polytomous response options (0, 1, 2, 3) of STAI “Form Y” to a dichotomous structure (0, 1) can be useful in clinical settings for adults aged >69 years (35). There have been substantial debates regarding whether multi-point response options should be used in the rating scales assessing elderly populations, such as patients with PD. A greater number of response categories in an ordinal scale allow for the capture of detailed information and distinction of minor clinical differences. However, an excessive number of response categories can induce confusion and fatigue in older patients, particularly if the investigator cannot make a clear distinction between each category or if the overall cognitive function of the patient is impaired (6, 9, 12). The recently developed GAI is one instance of employment of a dichotomous response category (“Agree” or “Disagree”) to indicate the level of anxiety in older adults (17).

We evaluated whether a reduction in the number of STAI response options would improve the measurement properties of the questionnaire in patients with PD. In the first instance, the rescored three-point STAI-trait demonstrated considerable improvement not only in polytomous category function, but also in Rasch model fit and scale targeting. The mean logit score was reduced on the person-item distribution map, indicating improvement in scale target deviation. Analysis of the gender-related DIF showed that item 35 “I feel inadequate” was easier for females to endorse. However, the DIF contrast was only marginally above the criterion, indicating that the gender DIF of item 35 was of minor concern. The benefit of reduction in the number of response options and conversion of the scale to a three-point Likert scale improved the polytomous response category function, scale targeting, and Rasch model fit without hindering the unidimensionality and separation reliability.

In contrast, although the rescored three-point STAI-state demonstrated an improvement in response category function and scale targeting, the number of items that did not fit the Rasch model (four misfitting items) or presenting gender-related DIF (two items) was similar to that of the original STAI-state. Therefore, additional modification (removal of items 7, 9, 14, and 18) was required in order to resolve the Rasch model misfit and gender DIF. Our revisions with removal of inappropriate items of the STAI-state subscale are in line with those of previous works proposing several shorter forms of the STAI-state composed of six or seven items (39–41). Although only four inappropriate items were removed from the STAI-state in the present study, future investigations in patients with PD should consider the assessment of clinimetric properties of several shorter versions of the STAI-state that have already been developed for clinical use.

As described above, the current analysis found that some of the original STAI items (STAI-state item 9 “I feel frightened” and item 18 “I feel confused,” STAI-trait item 25 “I feel like a failure” and item 35 “I feel inadequate”) were relatively less severe indicators of anxiety for male than for female. These gender-related DIFs were not virtually observed in prior Rasch analysis among non-PD population (34, 37). The discrepancy with the findings of previous reports could be due to the use of different versions of the instruments or to the different samples examined. The previous studies applied the first version of the STAI in relatively younger non-PD samples (34, 37). Moreover, STAI-state item 14 “I feel indecisive” and item 18 “I feel confused” exhibited high misfit to the Rasch model. This finding of misfitting items also differed from prior Rasch analysis studies of the STAI in non-PD samples (35, 36). While confusion or indecisiveness could be related to the overall anxiety levels of patients, they can also result from other non-motor symptoms such as apathy, fatigue, or depression in PD (42, 43).

The proposed amendment of the collapse of the four-category option to a three-category system in our study involves the application of odd numbers of response categories to offer other than even. An even or odd number of response options each have certain strengths and shortcomings to be considered. The rating scales that have an even number of response categories such as the original STAI can lead the patient to provide more distinct answers instead of simply providing a midpoint option in their odd counterparts (44). However, odd numbers of categories with midpoint options are known to resolve the questionnaire bias caused by forcing the respondents to provide an answer, and further studies are needed to elucidate the optimal number of response categories (44, 45).

There are limitations to the study that influence the generalizability of our findings. One such limitation of this study is its questionnaire-based design, which did not include patient interviews or clinical diagnosis of anxiety according to the Diagnostic and Statistical Manual of Mental Disorders criteria (1, 16). However, our study did not aim to diagnose specific anxiety disorders, and such patient interviews may not be appropriate for retrospective investigations such as this. Second, the subjects in this study were limited to *de novo* PD patients without any cognitive impairment at the time of PPMI study registration (21, 22). Our study was cross-sectional, using the PPMI online database among early and unmedicated patients, thus there was a limitation in the number of advanced stage PD patients. Floor effects of both subscales can be expected when utilizing the STAI in patients with early-stage PD with mild non-motor manifestations including neuropsychiatric and cognitive symptoms. Notably, anxiety levels appear to be too high in some early PD patients (17, 18). Given that the PPMI cohort has a relatively short duration after diagnosis (6.0 ± 3.6 months), it is possible that initial anxiety level may be partly explained as a psychological reaction to the diagnosis of their disease in some early-stage PD population (2, 46). Third, convergent validity was only tested against the MDS-UPDRS part 1 anxiety item and not against the other previously validated clinical rating scales for the measurement of anxiety in patients with PD. Fourth, our study did not assess the inter-rater or test-retest reliability. The test-retest reliability was demonstrated to be good for the STAI-trait but was not satisfactory for the STAI-trait in non-PD patients (3, 4). No such information is provided for PD patients and further studies are necessary to address its inter-rater and test-retest reliability.

## Conclusions

In conclusion, the rating scales that evaluate and quantify the severity of anxiety symptoms are particularly relevant for patients with PD. The STAI is one of the widely-used questionnaires for anxiety in clinical practice; however, the STAI has not been clinimetrically tested in patients with PD, based on modern test theories such as the Rasch measurement model. Our Rasch analysis complements previous findings based on the CTT and identified areas for possible amendments. Future studies should assess the clinimetric properties of the original version of STAI in broader populations including late-stage PD patients, and the harmonized amendments can be tested to assess the interpretation of items and ease of comprehension. We expect that the present study will substantially help to evaluate anxiety symptoms in patients with PD in both clinical research and clinical practice.

## Author Contributions

YK and H-JY participated in study design. J-HA, JuL, and WL interpreted data and contributed analytical tools. WL and H-JY acquired data and performed statistical analysis. J-HA, JuL, WL, and JiL contributed to writing of the manuscript and revised the manuscript for content. All authors read and approved the final manuscript.

### Conflict of Interest Statement

The authors declare that the research was conducted in the absence of any commercial or financial relationships that could be construed as a potential conflict of interest.
