# Supplementary material for: Measuring Anxiety in Patients With Early-Stage Parkinson's Disease: Rasch Analysis of the State-Trait Anxiety Inventory
Source: Front Neurol. 2019 Feb 13;10:49. doi: 10.3389/fneur.2019.00049 (PMC6383064; doi:10.3389/fneur.2019.00049)
Supplement: Supplementary file 1 [file Data_Sheet_1.docx]

Supplementary Material

Measuring anxiety in patients with early-stage Parkinson's disease: Rasch analysis of the State-Trait Anxiety Inventory

Hui-Jun Yang^1^, Joon-Ho Ahn^2^, Jungsun Lee^3^, Won Kee Lee^4^, Jiho Lee^5^, Yangho Kim^5,^*

*** Correspondence:** Yangho Kim, MD, PhD: [yanghokm@ulsan.ac.kr](mailto:yanghokm@ulsan.ac.kr)

| **Step 1: Unidimensionality**  • Assessed by a principal component analysis of the residuals  • At least ≥40% variance in the explained by the Rasch factor  • Unexplained variance attributable to the first two residual contrasts should be <10% with an eigenvalue for the first or second residual variances of <3.0 |
| --- |
| 🡫 |
| **Step 2: Item fit to the Rasch model**  • Investigated based on the mean-square (MnSq) value infit (the information-weighted fit) and outfit (the outlier-sensitive fit) statistics  • If the MnSq value of an item falls outside the range of 0.5–1.5 was considered as misfit to the Rasch model |
| 🡫 |
| **Step 3: Separation reliability for group comparisons**  • The separation reliability was assessed by the person separation reliability (PSR).  • Threshold value of the PSR would be >0.8 |
| 🡫 |
| **Step 4: Scale targeting**  • Visual inspection of the person-item distribution map  (Wright map) |
| 🡫 |
| **Step 5: Differential item functioning (DIF) analysis**  • Measured to examine the possibility that the clinical rating scales might function differently according to gender or age  • DIF was considered to be significant if DIF contrast above 0.60 logits difference |
| 🡫 |
| **Step 6: Response category function**  • Ordered threshold on the probability curves of the categories  • At least 10 observations in each response category  • Hierarchically ordered average measures and step calibrations  • The acceptable range of outfit MnSq value is < 2.0 |

**Supplementary figure 1.** A schematic representation of the Rasch analysis of the current study


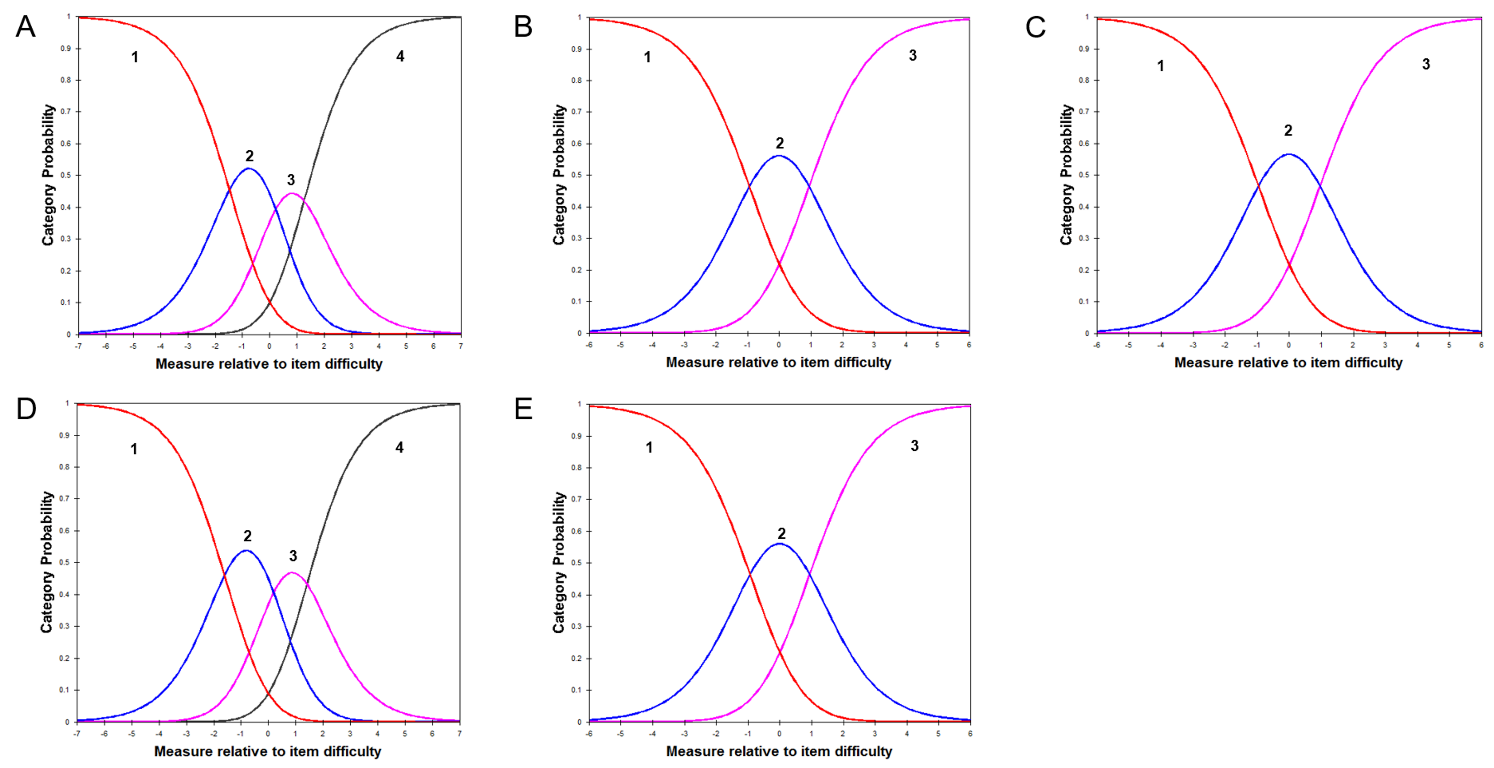


**Supplementary figure 2.** Probability curves of the State-Trait Anxiety Inventory subscales response categories demonstrate ordered thresholds in the study patients

(**A**) Category probability curve with the State-Trait Anxiety Inventory (STAI)-state (all 20 items) with four response categories (1, 2, 3, and 4). (**B**) Category probability curve with the rescored STAI-state with three response categories (1, 2, and 3). (**C**) Category probability curve with the shortened 16-item STAI-state with three response categories (1, 2, and 3). (**D**) Category probability curve for the STAI-trait (all 20 items) with four response categories (1, 2, 3, and 4). (**E**) Category probability curve for the STAI-trait with three categories (1, 2, and 3).
